# Effects of intradialytic exercise for advanced-age patients undergoing hemodialysis: A randomized controlled trial

**DOI:** 10.1371/journal.pone.0257918

**Published:** 2021-10-22

**Authors:** Hiroki Yabe, Kenichi Kono, Tomoya Yamaguchi, Yumiko Ishikawa, Yoshiko Yamaguchi, Hisanori Azekura

**Affiliations:** 1 Department of Physical Therapy, School of Rehabilitation Sciences, Seirei Christopher University, Mikatahara, Kita-ku, Hamamatsu, Shizuoka, Japan; 2 Department of Physical Therapy, International University of Health and Welfare, School of Health Sciences at Narita, Kozunomori, Narita, Chiba, Japan; 3 Department of Rehabilitation, Seirei Fukuroi Municipal Hospital, Kuno, Fukuroi, Shizuoka, Japan; 4 Department of Nursing, Sanaru Sun Clinic, Higashiiba, Naka-ku, Hamamatsu, Shizuoka, Japan; 5 Department of Nephrology, Sanaru Sun Clinic, Higashiiba, Naka-ku, Hamamatsu, Shizuoka, Japan; Universidade Estadual Paulista Julio de Mesquita Filho, BRAZIL

## Abstract

Previous reports have shown the benefits of intradialytic exercise to patients undergoing hemodialysis. However, most of those studies assessed the effects of exercise in middle-aged patients and little is known about advanced-age patients undergoing hemodialysis. Therefore, the present randomized controlled trial was performed to determine the effectiveness of exercise therapy in advanced-age patients undergoing hemodialysis. This non-blinded, randomized controlled parallel trial enrolled a total of 101 patients who were randomly assigned to intradialytic exercise (n = 51) or usual care (n = 50) groups. The training program included both resistance and aerobic exercises and was performed three times per week for 6 months. The aerobic exercise intensity was adjusted to a target Borg score of 13 for 20 minutes. Four types of resistance exercises were performed using elastic tubing, with three sets of 10 exercises performed at moderate intensity (13/20 on the Borg scale). The usual care group received standard care. Lower extremity muscle strength, Short Physical Performance Battery score, and 10-m walking speed were the outcomes and were evaluated before the hemodialysis session and after 6 months of training. There were statistically significant improvements in Short Physical Performance Battery score (effect size, 0.57; 95% confidence interval, 0.15‒1.95) in the exercise group relative to the control group. There were no statistically significant differences in lower extremity muscle strength or in the 10-m walking speed between the two groups. These findings suggest that 6 months of intradialytic training could improve physical function in older patients undergoing hemodialysis.

## Introduction

The aging of the general population has led to a worldwide increase in the number of patients undergoing hemodialysis [1]. In patients with chronic kidney disease (CKD), physical function declines as renal failure progresses [2]. These processes are accelerated in advanced-age patients undergoing dialysis because of the influences of both uremic conditions and aging [3]. Some previous studies have shown that low physical function is associated with mortality and other adverse events [4–6]. It is necessary that clinicians working in the fields of CKD and hemodialysis provide adequate care to older patients with low physical function.

Exercise therapy, a treatment for poor physical function in patients undergoing dialysis, has shown some evidence of effectiveness; however, there are also some limitations to this evidence. Some meta-analyses have indicated the effectiveness of exercise interventions on exercise tolerance [7], muscle strength [8], and quality of life [9]. A renal rehabilitation guideline established in 2019 recommended exercise therapy for patients undergoing hemodialysis at an evidence level of 1b [10]. Exercise therapy has been reported to improve muscle tissue adaptation by promoting physiological processes such as myogenesis, skeletal muscle regeneration, and regulation of myostatin mRNA activity [11]. The combination of aerobic exercise and resistance training as exercise therapy during dialysis leads to greater improvement in exercise tolerance than a single type of exercise [12]. However, most studies assessing the effects of exercise in patients undergoing hemodialysis have involved middle-aged patients and little is known about the effect of exercise in advanced-age patients undergoing hemodialysis. Older patients face an array of barriers to exercise, such as low self-efficacy, discomfort, disability, fear of injury, habits, environmental factors, cognitive decline, and fatigue [13]. One previous meta-analysis showed somewhat inconclusive evidence of the efficacy of exercise training for advanced-age patients undergoing hemodialysis because only one study in the meta-analysis reported on exercise therapy of advanced-age patients involving out-of-dialysis exercise [8]. The concepts of exercise intervention for young to middle-aged patients undergoing hemodialysis are not fully applicable to older patients, and whether exercise training improves physical function in older patients undergoing hemodialysis remains unclear.

Therefore, the present randomized controlled trial (RCT) was performed to determine the effectiveness of exercise therapy in advanced-age patients undergoing hemodialysis. To our knowledge, this is the first RCT to address this question. We hypothesized that intradialytic exercise would improve muscle strength, walking speed, and physical function of advanced-age patients and would be as effective in preventing physical function decline in advanced-age patients as in patients of other age groups.

## Materials and methods

### Study design and patients

This study was conducted at the outpatient hemodialysis facility of the Sanaru Sun Clinic (Hamamatsu, Japan). All patients who regularly attended the dialysis unit were evaluated for eligibility between April and May 2019. In this single-center, prospective, simple RCT, all patients were randomly assigned to either the exercise or the parallel-control group. Group allocation was performed using the sealed envelope method for all subjects. Random assignments were generated by an investigator who was not involved in the testing or training. Due to space limitations, patients from both groups remained in the same dialysis room, and therefore, it was not possible to blind patients and staff. All training programs were designed by a physical therapist, while the same nurse supervised the exercise and assessed the outcomes daily.

The inclusion criteria were as follows: age ≥ 70 years, a history of hemodialysis for the previous ≥6 months, and undergoing hemodialysis for 4 hours, three times per week. The exclusion criteria were the presence of a physical disability or severe orthopedic problems, a history of stroke within the past 6 months, recent hospitalization within the past 3 months, non-ambulatory status, dementia with an inability to perform exercise and assessment, and acute or chronic medical conditions that would preclude assessment of the outcome measures or performance of an exercise. Patients who were randomly assigned to the control group received the usual care but were given no instructions to exercise.

Ethical approval was provided by Seirei Christopher University (Approval No. 17-146-01), and written informed consent was obtained from all participants. The study complied with the World Medical Association Declaration of Helsinki. The authors confirm that this study is registered at the University Hospital Medical Information Network (UMIN 000038402).

### Exercise protocol

Patients randomized to the exercise group were offered 6 months of supervised, individually tailored exercise training three times per week. Each training session was performed during the first 2 hours of each hemodialysis session. The patients underwent hemodialysis and performed each exercise while in the supine position. Each training session began with a 5-minute warm-up period involving stretching exercises. Only lower-extremity exercises were performed to allow temporary vascular catheter access.

The aerobic exercise program specifically targeted aerobic capacity and consisted of ergometer cycling (TerasuErgoⅡ, ShowaDenki, Osaka, Japan) for 20 minutes. The exercise intensity was adjusted to a target Borg score of 13. The aerobic exercise protocols were consistent with previous publications [14, 15]. The following four types of resistance exercises were performed using an elastic tube (TheraBand Resistance Band Loops, THERABAND, Akron, OH, USA): leg extension, straight leg raise, hip abduction, and hip flexion. The exercise intensity was adjusted by the tube stiffness to achieve a target Borg score of 13. Three sets were performed, each consisting of 10 repetitions. The patients were instructed to complete each repetition with 5 seconds for concentric muscle actions and 5 seconds for eccentric muscle actions. The resistance exercise protocols were also performed as described in previous studies [14, 15]. However, because the facilities could not measure the one-repetition maximum, the intensity was adjusted on the Borg scale [16]. The nurse encouraged each patient during every exercise session and adjusted the training to maintain the rate of perceived exertion constant. The nurses assessed the risks in consultation with guidelines and previous literature [17], and monitored for exercise-related side effects and unintended effects during every exercise session.

The clinical characteristics obtained at baseline included age, sex, height, dry weight, body mass index, hemodialysis vintage, primary disease, medications, and laboratory data. Laboratory data (serum albumin, serum phosphorus, serum calcium, serum potassium, C-reactive protein, normalized protein catabolism rate, and hemoglobin) were obtained prior to HD. The Geriatric Nutritional Risk Index (GNRI) was calculated by incorporating serum albumin levels, body weight, and height, as follows:

GNRI=[14.89×albumin(g/dL)]+[41.7×(bodyweight/idealbodyweight)]

Body weight/ideal body weight was set to 1 when the patient’s body weight exceeded the ideal body weight [18].

Dialysis status was assessed based on the duration of dialysis, intradialytic weight gain, and standardized dialysis volume (Kt/V). Kt/V was used as an index of the amount of dialysis, and the value was calculated using the method described by Shinzato et al. [19].

### Outcomes

The lower extremity muscle strength (LES), Short Physical Performance Battery (SPPB) score, and 10-m walking speed were evaluated before and after 6 months of training. These measurements were performed before the hemodialysis session in the waiting room at the dialysis facility.

The LES was measured as the maximum voluntary isokinetic knee extensor strength, using a handheld dynamometer (Mobie; Sakai Medical Corp., Tokyo, Japan). The patients were seated on a chair in an upright posture with their hands on the sides of the chair and knees flexed at 90°. The dynamometer pad was placed perpendicular to the leg, immediately above the malleoli. The patients were instructed to push against the dynamometer pad by attempting to straighten their knees for a period of 5 seconds. The isokinetic knee extensor strength was measured twice on each side and the highest value for the right and left legs was used to calculate the LES.

The SPPB score has the following three components: balance, gait speed, and lower limb force. The score for each component ranges from 0 to 4 points. The final score is the sum of the three test scores which ranges from 0 to 12 points [20]. The 10-m walking speed was measured with the patients walking at a comfortable pace on a 14-m walking course (with 2 m added before and after the 10-m walking course).

### Statistical analysis

All data were inspected for normality with the Shapiro-Wilk test. Normally distributed data are presented as mean ± standard deviation and non-normally distributed data are presented as median and range. Baseline patient characteristics were compared using an unpaired *t*-test or chi-square test. Differences in the exercise groups between baseline and after 6 months of exercise therapy were determined with the paired *t*-test. The group effect was measured by analysis of covariance (ANCOVA) using the change in scores (Δ, post minus pre) as the dependent variable and the baseline value of the dependent variable in the model as a covariate. Additional covariates for the ANCOVA models were identified by comparison of group means and confidence intervals (CIs) at baseline to identify clinically meaningful differences in characteristics that were identified *a priori* as potential confounders (age and hemodialysis vintage) [21, 22]. Adjusted mean differences and 95% CIs are reported for between-group differences over time, expressed as the exercise group minus the control group. The effect size (ES) was calculated as the change in the exercise group minus the change in the control group, divided by the pooled standard deviation, corrected for sample size. Statistical analyses were performed using IBM SPSS Statistics for Windows ver. 24 (IBM Corp., Armonk, NY, USA).

## Results

**Fig 1:**
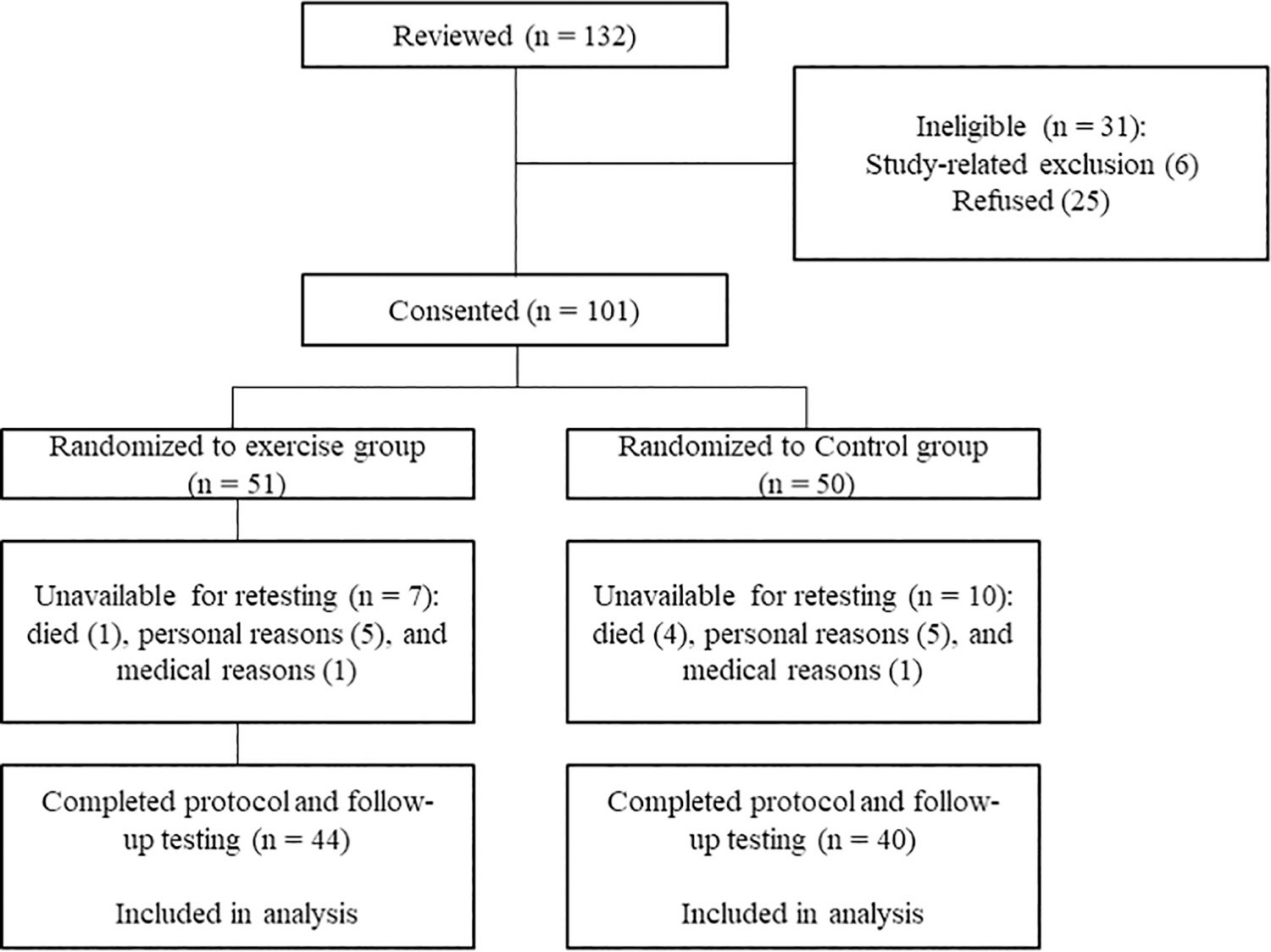
Flow of participants.

Among the 101 randomly assigned patients, 17 (16.8%) were unavailable for follow-up testing: 10 did not complete the final evaluations, two did not continue for medical reasons, and five died during the 6-month follow-up period (Fig 1). Thus, 84 patients (44 in the exercise group and 40 in the control group) were included in the analysis. There were no adverse events due to the intradialytic exercise.

The group comparison revealed no differences in any baseline characteristics between the two groups (Table 1). The SPPB score (10 [7.0‒11.3] points at baseline; 12 [8.6‒12] points at 6 months, p < 0.05) improved in the exercise group. Conversely, no significant changes were observed in the control group. No significant difference was observed in the LES or the 10-m walking speed between the two groups at baseline or after an intervention.

**Table 1:** Patient characteristics.

	All (n = 84)	Control (n = 40)	Exercise (n = 44)	p
Age (year)	78.8 ± 6.4	79 ± 6.7	78.7 ± 6.3	0.66
Sex (men/women [n]))	47/37	21/19	26/18	0.53
Height (cm)	155.3 ± 9.9	155.2 ± 10.2	155.4 ± 9.8	0.93
Dry weight (kg)	51.7 ± 9.4	51.3 ± 8.8	52.1 ± 10	0.71
BMI (kg/m^2^)	21.3 ± 2.7	21.3 ± 2.8	21.4 ± 2.6	0.82
HD vintage (month)	66.2 ± 70.9	70.9 ± 79.2	61.9 ± 63	0.75
Disease (n [%])				
Nephrosclerosis	35 (41.7)	16 (40)	19 (43.2)	0.76
Diabetes	21 (25)	8 (20)	13 (29.5)	0.31
Chronic glomerulonephritis	19 (22.6)	11 (27.5)	8 (18.2)	0.31
Others	9 (10.7)	5 (12.5)	4 (9.1)	0.61
Medication (n [%])				
Ca antagonist	29 (34.5)	10 (25)	19 (43.2)	0.08
ARB	25 (29.8)	9 (22.5)	16 (36.4)	0.16
ACE inhibitor	0 (0)	0 (0)	0 (0)	-
β blocker	14 (16.7)	4 (10)	10 (22.7)	0.12
Laboratory data				
Alb (g/dL)	3.7 ± 0.3	3.7 ± 0.3	3.8 ± 0.3	0.28
GNRI	96.4 ± 7.1	95.8 ± 6.7	96.9 ± 7.6	0.50
P (mg/dL)	5.4 ± 1.5	5.4 ± 1.6	5.3 ± 1.3	0.67
Ca (mg/dL)	8.5 ± 0.8	8.6 ± 0.8	8.5 ± 0.8	0.78
K (mEq/L)	4.6 ± 0.7	4.7 ± 0.6	4.5 ± 0.7	0.10
nPCR	0.9 ± 0.2	0.9 ± 0.2	0.9 ± 0.2	0.91
Kt/V	1.5 ± 0.3	1.5 ± 0.3	1.5 ± 0.3	0.64
Hb (g/dL)	10.8 ± 0.9	10.7 ± 0.9	10.9 ± 1	0.33
CRP (mg/dL)	0.5 ± 1.3	0.8 ± 1.8	0.3 ± 0.4	0.10
Vascular acsess (n[%])				
AVF	73(86.9)	33(82.5)	40(90.9)	0.34
AVG	11(13.1)	7(17.5)	4(9.1)	

BMI: body mass index; HD: hemodialysis; ARB: angiotensin receptor blocker; ACE: angiotensin converting enzyme; Alb: serum albumin; GNRI: geriatric nutritional risk index; P: serum phosphorus; Ca: serum calcium; K: serum potassium; nPCR: normalized protein catabolic rate; Hb: hemoglobin; CRP: C‒reactive protein; AVF, arteriovenous fistula; AVG, arteriovenous graft.

Mean ± standard deviation.

For the comparison between the exercise and control groups before and after the intervention, and because of potentially clinically important differences in age and hemodialysis vintage between the two groups, these variables were included as covariates in all ANCOVA models of absolute change scores [21, 22]. Statistically significant increases in the ΔSPPB score were found in the exercise group (0.7 ± 2.1) compared to the control group (−0.4 ± 2.0), with a moderate ES of 0.57 (Table 2). The ΔLES and Δ10-m walking speeds were not significantly different between the two groups and the ES were 0.18 and 0.00, respectively.

**Table 2:** Comparison between exercise and control groups before and after an intervention.

	Control				Exercise				Adjusted mean difference (95% CI)	Effect size	p
	pre	post	p	Δ	pre	post	p	Δ			
LES	17.4 ± 7.8	15.2 ± 6.8	0.80	0.3 ± 4.1	16.7 ± 8.9	16.7 ± 8.9	0.69	-0.6 ± 5.6	0.94 (-1.22 to 3.09)	0.18	0.64
10-m walking speed	1.1 ± 0.5	1.1 ± 0.4	0.89	0 ± 0.4	1.1 ± 0.3	1.1 ± 0.3	0.78	0 ± 0.2	-0.02 (-0.15 to 0.12)	0.00	0.54
SPPB	10 (7.0‒12.0)	9 (7.0‒11.0)	0.12	0 (-1.5 to 0)	10 (7.0‒11.3)	12 (8.6‒12.0)†	0.04	1 (0‒2.0) †	1.05 (0.15‒1.95)	0.57	0.01*

Values are expressed as mean ± SD or median (interquartile range) according to normality by Shapiro-Wilk test. CI, confidence interval; LES, lower extremity muscle strength; SPPB, Short Physical Performance Battery; Δ, 6-month value—baseline value.

†: Significant difference between the pre and post intervention by Wilcoxon signed-rank sum test (p < 0.05).

*: Significant difference between the control group by ANCOVA (p < 0.05).

## Discussion

In this evaluation of the effect of intradialytic exercise in older patients, the primary finding was that 6 months of intradialytic aerobic and resistance training significantly improved the physical function of advanced-age patients.

It is particularly important to know whether such interventions are effective in advanced-age patients and not only in middle-aged patients, because of the aging of society. In 2019, the renal rehabilitation guidelines from the Japanese Society of Renal Rehabilitation recommended exercise therapy for patients undergoing hemodialysis at an evidence level of 1b [10]. In 2016, the European Renal Best Practice Guideline Development Group published new clinical practice guidelines for advanced-age patients with CKD [23]. These new practice guidelines recommended assessment of and intervention for low physical function in advanced-age patients with CKD. A previous study showed that hospitalization-free survival was lower in a group of patients who completed a 6-month exercise trial than in the control group [24]. However, a previous meta-analysis showed somewhat inconclusive results on the effectiveness of exercise training in older versus younger people, because no studies assessed the association between exercise training and exercise tolerance in advanced-age people undergoing hemodialysis. Because the present study is one of the few RCTs in this field, it contributes to the accumulation of evidence regarding the effects of exercise therapy in older patients undergoing dialysis.

The SPPB score is a good indicator of the change in physical function in older patients and can also be applied to older patients undergoing dialysis. A previous study showed that the minimal detectable change (MDC) at the 95% CIs of the SPPB score in community-dwelling older adults was 0.8 (95% CI, 0.88–0.95) [25]. The minimal clinically important difference (MCID) of the SPPB score in community-dwelling older persons showed that the change in score corresponding to a small and moderate ES was 0.54 and 1.34 points, respectively. The difference in the SPPB score in patients with a small decline vs. those without a small decline ranged from 0.27 to 0.55 points, whereas the difference in the SPPB score for a substantial decline ranged from 0.99 to 1.88 points [26]. In a study of patients undergoing hemodialysis, the MDC of the SPPB score for the 95% CI was 1.7 points, and the reliability of the SPPB score was considered acceptable [27]. The ΔSPPB score in the exercise group in the present study was lower than the previous values of MDC or MCID; however, the ES in the present study suggested a clinically significant improvement in physical function as compared with these previous values.

Poor physical function, as evaluated by the SPPB score, may be associated with a worse prognosis. Exercise therapy is clinically important for patients undergoing hemodialysis. The SPPB score is reported to be a useful predictor of acute illness and subsequent physical dysfunction, readmission, and death in community-dwelling older persons [28, 29]. In a study of 542 healthy community-dwelling persons aged >65 years, 84 (15.5%) lost the ability to walk 400 m within a 3-year follow-up period, which was indicated by a cut-off SPPB score of <9 points [30]. To date, no study has focused on the effects of exercise therapy on improving the prognosis of patients undergoing hemodialysis. However, one study of patients undergoing hemodialysis showed no change in frail patients from an SPPB score of ≤6 to >6 points and a 29.3% shift in non-frail patients from an SPPB score of >6 to ≤6 points after a 12-month follow-up period [27]. Improvement in physical function by exercise therapy may improve the prognosis of advanced-age patients undergoing dialysis; at a minimum, it may prevent the decline in physical function associated with sedentary lifestyle.

Exercise therapy improves physical function in older patients undergoing dialysis, but the mechanism of benefit may differ from that in middle-aged patients. Previous studies have indicated that exercise therapy for middle-aged hemodialysis patients improved muscle strength [8, 31] and the 10-m walking speed [32]. The effect of exercise therapy for advanced-age patients undergoing hemodialysis, as compared with the effect on middle-aged patients, differs across studies. Some studies of older patients undergoing hemodialysis showed a significant effect of exercise on LES [33, 34], whereas others did not [35, 36]. Exercise tolerance was also significantly affected in some studies [33, 37], but not in others [35], Yamaguchi et al. [38] showed, in a non-RCT, that a 6-month intradialytic exercise program for patients (mean age, 69.1 ± 7.6 years) with low physical function (SPPB score <9 points) significantly improved their SPPB scores. Chen et al. [34] showed the effect of exercise on the SPPB score but not on the 10-m walking speed in an RCT of a 4-month intradialytic resistance exercise program for advanced-age patients (mean age, 71.1 ± 12.6 years). These studies support the findings of the present study with respect to a significant effect on the SPPB score, but little effect on the LES and 10-m walk speed in older patients undergoing hemodialysis. The mechanisms of these differences are unclear, but several factors, such as age, hemodialysis vintage, exercise dose (exercise duration, intensity, and time), type of exercise, nutritional state, hemodialysis dose, and uremic state, may be related to the effect of exercise; therefore, further studies are needed.

No serious adverse events were noted during 6 months of intradialytic exercise in elderly patients. A previous review on exercise in patients undergoing hemodialysis revealed that 13 of the 29 reviewed studies specifically reported no serious complications resulting from participating in a prescribed exercise intervention [39]. Moreover, three studies [21, 34, 39] and one meta-analysis [40] reported no statistically significant differences in adverse events between the exercise and control groups. Taken together with the results of this study, this suggests that intradialytic exercise can be safely performed in elderly patients on hemodialysis requiring risk management.

This study had some limitations. First, the findings may not be fully generalizable due to its single-center design. Because this was a single-center study with a fixed number of study participants, we did not calculate the sample size first. Sample size calculations (α = 0.8, β = 0.05, and ES = 0.8) revealed this number to be 34 for each group. As a result, the number of subjects was sufficient, but the sample size should have been calculated when the research was planned. Our findings that LES and the 10-m walking speed could not be improved in a period of 6 months suggest the need for studies with long-term interventions, preferably performed at multiple centers. Moreover, this study excluded patients who could not walk independently; therefore, we included elderly patients with a relatively high physical function. The effect of exercise therapy on elderly patients with low physical function requires further study. Second, there may have been some bias caused by the randomization and blinding procedures in this study. The envelope method of randomization may not be optimal in ensuring allocation concealment and block randomization may have been preferable. This study could not blind patients and evaluators; thus, there may have been implementation and observer biases. The case reduction bias may also be high due to a lack of intention-to-treat analysis. The lack of a sham exercise control activity, due to the single study locale, is another limitation. Finally, this study did not measure patient quality of life. Further studies on whether exercise therapy improves the quality of life in elderly dialysis patients through improved physical function are needed.

## Conclusion

In this study, we found that after 6 months of intradialytic aerobic and resistance training, we observed statistically significant increases in the ΔSPPB score among a group of older adults, indicating that exercise therapy improves physical function in older patients undergoing hemodialysis. Although the effectiveness may differ from that in middle-aged patients, this change is clinically important in older patients and thus should be considered with relevant safety precautions in older patients undergoing dialysis.

## Supporting information

S1 Checklist CONSORT checklist. (DOC)

S1 File Study protocol. (DOCX)
