# Supplementary material for: Effects of intradialytic exercise for advanced-age patients undergoing hemodialysis: A randomized controlled trial
Source: PLoS One. 2021 Oct 22;16(10):e0257918. doi: 10.1371/journal.pone.0257918 (PMC8535393; doi:10.1371/journal.pone.0257918)
Supplement: S1 File — (DOCX) [file pone.0257918.s002.docx]

倫　理　審　査　申　請　書

聖隷クリストファー大学

倫理委員会委員長　様

申　請　者

所　属　　聖隷クリストファー大学

学籍番号(院生)

氏　名　　　　矢部　広樹　　　　印

指導教員(申請者が大学院生の場合)

所　属

氏　名　　　　　　　　　　　　　印

| １　研究課題名  高齢の血液透析患者に対する透析中の運動療法の効果に関する研究：ランダム化比較試験 | | | | | |
| --- | --- | --- | --- | --- | --- |
|  | | | | | |
| 氏名 | 所属 | 受講済講習会等の種類※ | 分担業務の内容 | CITI Japan eラーニング修了証番号 | |
|  |  |  |  | RCR（No）／修了日 | HSR（No）／修了日 |
| ２　研究責任者  　　矢部広樹 | 聖隷クリストファー大学 | ☑CITI eﾗｰﾆﾝｸﾞ  ☑講習会/研修会 | ☑研究業務全般  ☐( ) | ＃6576446  西暦 2016年 8月 1日 | ＃7228378  西暦 2016年 8月 2日 |
| ３　研究分担者 |  | ☐CITI eﾗｰﾆﾝｸﾞ  ☐講習会/研修会 | ☐研究業務全般  ☐( ) | ＃  西暦 　 年 　月 　日 | ＃  西暦 　 年 　月 　日 |
|  |  | ☐CITI eﾗｰﾆﾝｸﾞ  ☐講習会/研修会 | ☐研究業務全般  ☐( ) | ＃  西暦 　 年 　月 　日 | ＃  西暦 　 年 　月 　日 |
|  |  | ☐CITI eﾗｰﾆﾝｸﾞ  ☐講習会/研修会 | ☐研究業務全般  ☐( ) | ＃  西暦 　 年 　月 　日 | ＃  西暦 　 年 　月 　日 |
|  |  | ☐CITI eﾗｰﾆﾝｸﾞ  ☐講習会/研修会 | ☐研究業務全般  ☐( ) | ＃  西暦 　 年 　月 　日 | ＃  西暦 　 年 　月 　日 |
|  |  | ☐CITI eﾗｰﾆﾝｸﾞ  ☐講習会/研修会 | ☐研究業務全般  ☐( ) | ＃  西暦 　 年 　月 　日 | ＃  西暦 　 年 　月 　日 |
|  |  | ☐CITI eﾗｰﾆﾝｸﾞ  ☐講習会/研修会 | ☐研究業務全般  ☐( ) | ＃  西暦 　 年 　月 　日 | ＃  西暦 　 年 　月 　日 |
|  |  | ☐CITI eﾗｰﾆﾝｸﾞ  ☐講習会/研修会 | ☐研究業務全般  ☐( ) | ＃  西暦 　 年 　月 　日 | ＃  西暦 　 年 　月 　日 |
|  |  | ☐CITI eﾗｰﾆﾝｸﾞ  ☐講習会/研修会 | ☐研究業務全般  ☐( ) | ＃  西暦 　 年 　月 　日 | ＃  西暦 　 年 　月 　日 |
|  |  | ☐CITI eﾗｰﾆﾝｸﾞ  ☐講習会/研修会 | ☐研究業務全般  ☐( ) | ＃  西暦 　 年 　月 　日 | ＃  西暦 　 年 　月 　日 |

| ４　研究の概要  　身体機能や動作能力が低下した高齢透析患者に対するリハビリテーションは、日本国内だけでなく、全世界的に不十分な現状にある。透析患者は、蛋白エネルギー消耗状態（protein energy wasting: PEW）にある。加えて、近年の透析患者は高齢化が進み、虚弱、不活動によって廃用状態を呈しており、PEWとともに尿毒症性サルコペニアという概念も提唱され、骨格筋萎縮が進行し、全身の筋力や筋肉量が低下し、身体パフォーマンスも重度に低下しているとされている。  　透析治療中に行う運動療法は、それ以外の運動形態よりも継続率が高く、運動時間の確保が難しい患者において効果的とされる。我々が行ったメタアナリシスによって、透析治療中に行う有酸素運動やレジスタンストレーニングは、筋力や運動耐容能を向上させることが明確なエビデンスとなっている^1)^。しかしながら、このメタアナリシスで抽出した全ての無作為化比較試験（randomized contolled trial: RCT）は、年齢が若く、身体機能の保たれた透析患者を対象にしている。実際に、2017年に行われた高齢血液透析患者に対する運動療法のメタ解析では、高齢者透析患者に対する運動療法に関するRCTは1件しか該当しなかった（Matsuzawa 2017)。現在、高齢の血液透析患者に対して、透析治療中に実施する運動療法が、身体機能を向上させるか否かは、明らかになっていない現状である。  　2012年の日本透析医学会統計調査委員会の報告によると、本邦の透析患者は男女とも75歳～80歳の患者層が最も数が多く、75歳以上の透析患者はADLに何らかの介助を必要とする者が多い。さらに高齢透析患者では転倒の発生件数が多く、骨が脆弱化しているため容易に骨折しADLが一層低下するといった悪循環に陥りやすい。この悪循環を断ち切るためには、筋力や、動作能力を改善させるための取り組みを実践すべきであり、高齢透析患者に対するリハビリテーションのエビデンスの構築が喫緊の課題である。  　そこで本研究では、高齢血液透析患者に対して、透析治療中のレジスタンス運動と有酸素運動を実施することで、身体機能を改善させることができるかどうか、検討することを目的とした。   - 研究目的   　透析治療中に行う運動療法が、高齢透析患者の筋力と筋肉量、身体機能の向上に効果があるか否かを、RCTにて検証する。   - 研究の方法 - 臨床研究実施施設：さなるサンクリニック - 臨床研究の期間：倫理委員会承認後〜1年間 - デザイン：RCT(並行比較試験) - 対象   維持血液透析患者100名程度(介入群50名、対照群50名)。介入群と対照群は、基準を満たす患者の中から、ランダムに割り当てる。以下に対象の取り込み基準と除外基準を示す。  ＜取り込み基準＞   - 70歳以上 - 週3回4時間透析を実施 - 通常の透析治療が安定して実施できている - 研究参加に理解が得られる認知機能とコミュニケーション能力がある - 主治医から研究参加に関する許可が得られる   ＜除外基準＞   - 血液透析導入後6カ月未満の患者 - 研究参加に同意が得られない患者 - 安定しない心血管疾患の存在 - 十分にコントロールされていない急性疾患の存在 - コミュニケーションが取れない認知機能障害や脳血管疾患の存在 - 安定しない血液透析治療（透析関連低血圧、除水困難等）の存在 - 主治医の許可が得られない場合 - 介入方法   　介入群は、通常の透析治療に加え、週3回の透析前半2時間の内に、準備体操の後、ゴムチューブを用いたレジスタンス運動と、簡易エルゴメータ(てらすエルゴ TE2-20)を用いた有酸素運動を実施する。運動介入中は、血圧、心拍数と自覚的疲労度をモニタリングする。対照群は、通常の透析治療を実施する。介入期間は6カ月間とする。  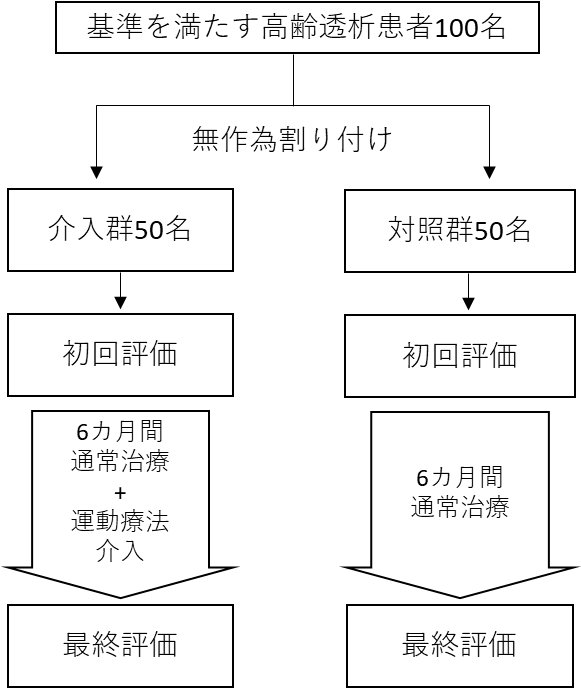  図　研究の流れ  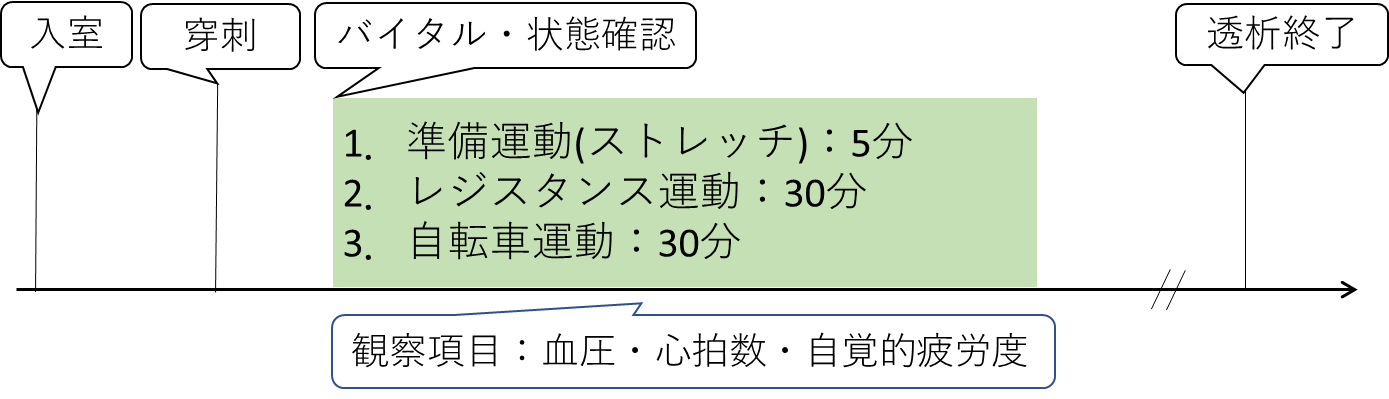  図　介入群の運動プロトコル   - 準備体操(5分)   股関節屈曲、内外転・内外旋、膝関節屈伸、足関節底背屈について、セルフストレッチを実施する。運動は各運動共に10回×1set実施する。  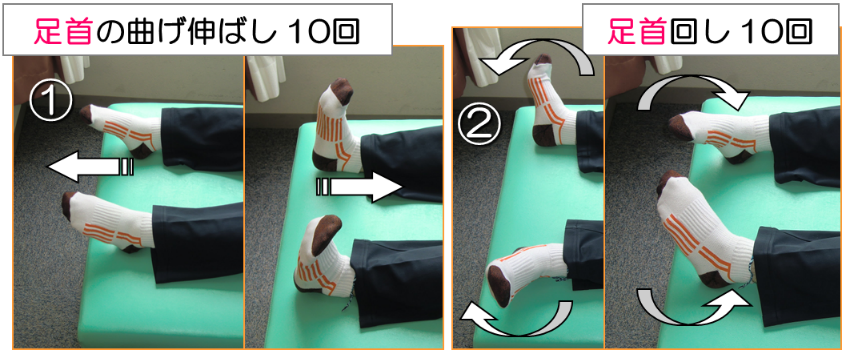  図　準備体操の例   - レジスタンス運動(30分)   　チューブを両足とベッドやチェアに固定、もしくは自身で把持し、チューブの抵抗を利用して筋に負荷をかけて実施する。膝の伸展、下肢の内転・外転、股関節の屈曲等、足関節底屈、股関節伸展を実施する。回数・セット数は、各運動共に10回×3setを目標に、10回×1setから開始し、週を重ねる毎に徐々にセット数を増加させる。強度は自覚的疲労度の13とする。  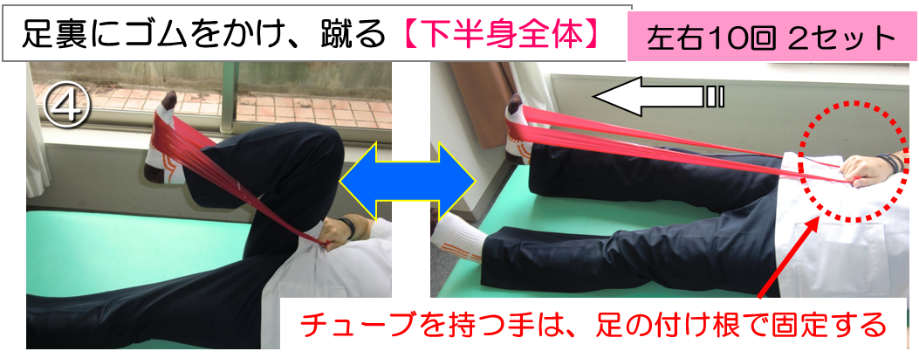  図　レジスタンス運動の一例   - 有酸素運動   強度は、自覚的疲労度の13(ややきつい)程度とし、無負荷での5分間の準備運動の後、20分間実施する。運動は自覚的疲労度の11(楽である)から開始し、週ごとに強度を増加させて実施する。  図　有酸素運動の様子   - 測定項目   メインアウトカムとして、身体機能として、握力、膝伸展筋力、10m歩行速度、Short physical Performance Battery(SPPBを測定する。握力はデジタル握力計(T.K.K5401, 竹井機器)を用いて立位にて測定し、左右2回測定し最大値を記録する。膝伸展筋力は徒手筋力計(Mobie 酒井医療)にて左右2回測定し、最大値を記録する。  その他、基本情報としてカルテより身長、体重、ドライウェイト、既往歴、服薬状況、血液検査所見を採集する。血液検査所見は、Alb、CRP、intactPHT、GA、GNRI、P、β2MG、Kt/V、Hb、Ca、HbA1c、nPCRとする。カルテ情報は、毎月定期的に評価される値を用いる  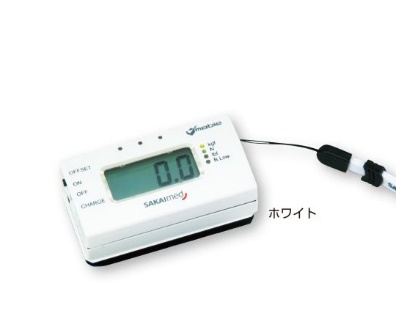  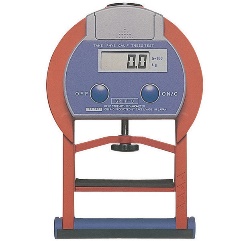  徒手筋力計  Mobie TK-11538  デジタル握力計  T.K.K5401  図2　測定機器  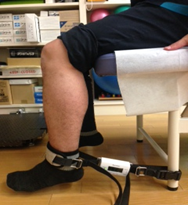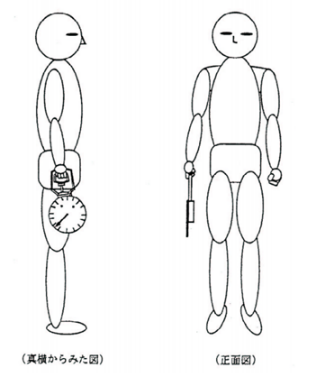  膝伸展筋力  握力  図3　測定の様子  ・　研究施設と測定場所  　さなるサンクリニックの診察室および透析室にて実施する。   - 研究の組織体制   研究代表者の矢部広樹は、研究計画の立案から研究進捗の確認、介入方法の立案、全体のデータの解析、論文執筆までを責任をもって実施する。  研究協力者は、研究のリスクマネジメント、研究のデータ測定と、運動療法の実施、またデータ解析や論文執筆の助言を行う。  表1 研究協力体制と役割   \| 役割 \| 氏名 \| 所属、役職、職種 \| 研究の分担 \| \| --- \| --- \| --- \| --- \| \| 代表者 \| 矢部広樹 \| 聖隷クリストファー大学、助教、理学療法士 \| 研究計画の立案、研究の進捗確認、データ解析、論文執筆 \| \| 協力者 \| 畦倉久紀 \| さなるサンクリニック、理事長、医師 \| リスクマネージメント \| \| 協力者 \| 山口慶子 \| さなるサンクリニック、総看護師長、看護師 \| データ測定、運動療法の実施 \| \| 協力者 \| 石川有美子 \| 名港共立クリニック　看護主任、看護師 \| データ測定、運動療法の実施 \| \| 協力者 \| 河野健一 \| 国際医療福祉大学、講師、理学療法士 \| データ解析の補助、論文執筆の補助 \|   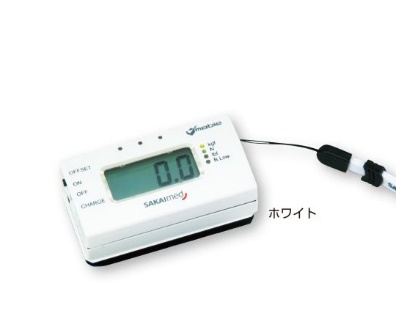  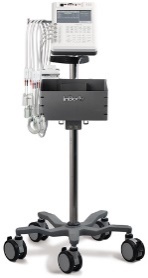  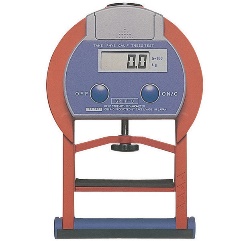  徒手筋力計  Mobie TK-11538  デジタル握力計  T.K.K5401  体組成計  Inbody S10  図2　測定機器  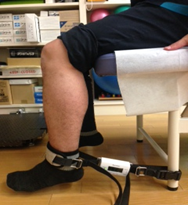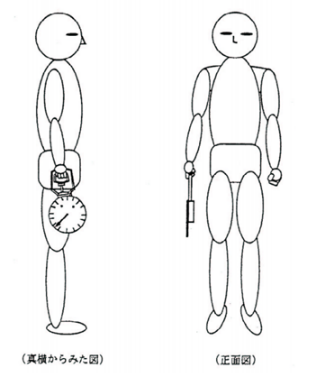  握力  身体組成  膝伸展筋力  図3　測定の様子  ・　研究施設と測定場所  　さなるサンクリニックの診察室および透析室にて実施する。   - 研究体制と役割分担   研究責任者の矢部広樹は、研究計画の立案から研究進捗の確認、介入方法の立案、全体のデータの解析、論文執筆までを責任をもって実施する。  　研究協力者は、研究のリスクマネジメント、研究のデータ測定と、運動療法の実施、またデータ解析や論文執筆の助言を行う。  　表1 研究協力体制と役割   \| 役割 \| 氏名 \| 所属、役職、職種 \| 研究の分担 \| \| --- \| --- \| --- \| --- \| \| 責任者 \| 矢部広樹 \| 聖隷クリストファー大学、助教、理学療法士 \| 研究計画の立案、研究の進捗確認、データ解析、論文執筆 \| \| 協力者 \| 畦倉久紀 \| さなるサンクリニック、理事長、医師 \| リスクマネージメント \| \| 協力者 \| 山口慶子 \| さなるサンクリニック、総看護師長、看護師 \| データ測定、運動療法の実施 \| \| 協力者 \| 石川有美子 \| さなるサンクリニック　看護主任、看護師 \| データ測定、運動療法の実施 \| \| 協力者 \| 河野健一 \| 国際医療福祉大学、講師、理学療法士 \| データ解析の補助、論文執筆の補助 \| | | | |
| --- | --- | --- | --- | --- | --- | --- | --- | --- | --- | --- | --- | --- | --- | --- | --- | --- | --- | --- | --- | --- | --- | --- | --- | --- | --- | --- | --- | --- | --- | --- | --- | --- | --- | --- | --- | --- | --- | --- | --- | --- | --- | --- | --- | --- | --- | --- | --- | --- | --- | --- | --- |
| ５　研究の対象及び選定方法  研究協力施設である、さなるサンクリニックの維持血液透析患者100名程度とし、取り込み基準は下記の通りとする。  ＜取り込み基準＞   - 70歳以上 - 週3回4時間透析を実施 - 通常の透析治療が安定して実施できている - 研究参加に理解が得られる認知機能とコミュニケーション能力がある - 主治医から研究参加に関する許可が得られる   尚、取り込み基準に合致した透析患者であっても、運動療法を始める際は、毎回、臨床の責任者から許可を取るものとする。  ＜除外基準＞   - 血液透析導入後6カ月未満の患者 - 研究参加に同意が得られない患者 - 安定しない心血管疾患の存在 - 十分にコントロールされていない急性疾患の存在 - コミュニケーションが取れない認知機能障害や脳血管疾患の存在 - 安定しない血液透析治療（透析関連低血圧、除水困難等）の存在 - 主治医の許可が得られない場合   ＜対象者に同意を得るまでの方法＞   1. 主治医に取り込み基準・除外基準を説明し、候補者を選定するように依頼する（資料⑤）。 2. 主治医は、候補者に対して口頭と書面（資料②）にて研究について説明する。その際に、主治医からも研究協力については自由意志であり、参加同意の撤回はいつでも可能であり、参加に同意されない場合や中断された場合にも、何も不利益や支障は生じないことを十分に説明していただく。また患者から研究に関する説明を担当者からしても良いかの同意を得る。 3. 対象者として基準を満たし、かつ主治医からの許可と、説明に対する本人の承諾が得られた患者に対し、研究の依頼を、研究責任者か、研究協力者が、患者本人へ口頭および書面(資料①②)にて実施する。説明は透析室にて実施し、説明後に了承が得られた場合は同意書(資料③)と同意撤回書（資料④）を手渡す。 4. 同意書は、説明を理解した上で考慮時間を設け、氏名、同意年月日を記載の上、クリニック受付に設置した回収BOXにて、1週間以上の考慮期間を設け、後日回収する。同意書は、対象者用と研究者用に2通作成し，それぞれが一通ずつ保管する。同意書は封筒に入れ、個人情報を保護する形で回収Boxに回収する。   ＜実施場所＞  さなるサンクリニック  研究の説明：診察室　透析室  運動療法の実施：透析室  　　データの測定：透析室前の待合室 | | | |
| ６　研究による利益・不利益等  ＜利益＞  対象者は身体機能評価を受けることで、自身の身体機能の現状を知ることができる。また運動療法を行うことで、自身の身体機能を向上させることができる。  ＜不利益＞  本研究の不利益は、運動療法の実施による多少の疲労感と時間的拘束、また身体機能評価に必要な時間的拘束と、評価による多少の努力と疲労感を必要とする点が挙げられる。時間的拘束としては、1回の運動には準備から終了まで60分程度かかり、週3回6カ月継続する。運動機能評価は、1回30分程度かかる。 | | | |
| ７　申請の理由  下記の項目について、倫理的配慮が十分かどうか審査して頂きたい   - 群分けによる研究実施における患者への配慮は適切か - 研究対象者に理解を求める方法は適切か - 研究対象者に同意を得る方法は適切か - 個人のプライバシーや個人情報は適切に管理できているか - 安全性は適切に担保されているか | | | |
| ８　研究における倫理的配慮   - 群分けによる研究実施における患者への配慮は適切か   本研究は、通常治療のみを行う対照群を設定しているが、対照群には6か月後の研究期間終了後に、希望があれば介入群と同等の運動療法介入ができる旨を、口頭及び書面にて説明し、運動により得られる効果は同等である旨を説明する   - 研究対象者に理解を求める方法は明確にされているか   研究対象者には、あらかじめ主治医より、研究分担者が研究に関する説明をしてもよいかの同意を確認する。研究の説明の同意が得られた場合は、説明文書(資料①)と依頼文書(資料②)を用いて、研究参加の依頼を行う。説明文書では、研究の目的に加え、実際に研究対象者へお願いする事項として、研究の流れ、運動と評価の方法、運動の安全性、その他について具体的に説明する。説明文書は、図表やイラスト、平易な用語を用いて、対象者の理解が得られやすいように工夫して説明する。依頼文書では、研究の目的、方法、利益と不利益、予測される不利益に対する対策、研究参加の自由意志と同意の撤回、個人情報の取り扱い、研究成果の公表、研究に関する質問窓口について、それぞれ口頭および書面にて説明する。   - 研究対象者に同意を得る方法が明確にされているか  1. 研究対象者への同意は、下記の順序で得る 2. 主治医が対象者に対して研究に関する説明をしてもよいかの同意を得る。 3. 研究の説明に対する本人の承諾が得られた患者に対し、研究責任者と協力者が患者本人へ研究の説明を口頭および書面(資料①②)にて実施し、同意が得られた場合は同意書(資料③)と同意撤回書（資料④）を手渡す。 4. 同意書は、説明を理解した上で考慮時間を設け、氏名、同意年月日を記載の上、クリニック受付に設置した回収BOXにて、1週間以上の考慮期間を設け、後日回収する。同意書は封筒に入れ、個人情報を保護する形で回収Boxに回収する。 5. 同意書は、対象者用と研究者用に2通作成し，それぞれが一通ずつ保管する。   同意撤回書は、下記の手順で処理する。   1. 研究の説明の際に、研究参加の同意はいつでも撤回できる旨を説明し、同意書と合わせて同意撤回書（資料④）を手渡す。 2. 同意撤回書は、クリニック受付に設置した回収BOXにて随時回収する。同意書は封筒に入れ、個人情報を保護する形で回収Boxに回収する。 3. 研究協力者は回収BOXを毎朝確認し、同意撤回書を回収・受理する。 4. 同意撤回書は対象者用と研究者用に2通作成し，それぞれが一通ずつ保管する。 5. 同意撤回書が提出された対象者のデータは、研究責任者が、紙媒体のものはシュレッダーにて、電子媒体のものもパソコン内にデータが残らない形で全て削除する。  - 個人のプライバシーや個人情報は守られているか   　研究において知り得たデータは、個人情報として厳重に取り扱い、研究データは個人が特定できない形で処理する。測定データは匿名化の後、データベースへ記載する。対応表は評価結果の用紙と共に全て鍵付きの保管庫で管理し、研究開始日より5年間厳重に保存する。各施設の学会等で研究結果を発表する際は個人が特定できないように提示する。評価結果の電子データはパスワードをかけて状態で管理する。電子データは専用のパソコンおよび専用の外部記憶メディアで管理し、パソコンはオンライン環境から常時切り離して管理する。データを破棄する場合は、紙媒体はシュレッダーにて、電子データはデータ末梢ソフトを用いてパソコン内にデータが残らない形で消去する。研究協力者は、本研究の専用パソコンからのみデータを閲覧できるものとし、データを複写して利用しないようにする。   - 安全性に対する配慮がなされているか   　透析中に実施する運動療法は、循環動態が安定する透析の前半2時間で行えば、安全に実施可能であることが示されている^1)^。また、透析患者に対する運動療法は、アメリカスポーツ医学会のガイドラインで推奨されており、今回の研究で実施する運動プログラムも、アメリカスポーツ医学会等で透析患者や高齢者に対する運動療法の方法として推奨されている方法であり、安全に実施が可能である。もし異常所見の出現や、患者自身の中止の申し出があった場合は、速やかに運動は中止する。さらに、異常事態発生に備えて救急用品も常備する。また主治医が院内に勤務し、かつ直ぐに連絡が取れる体制で実施するものとする。  研究で用いる身体機能評価は、老年医学の分野で確立され、かつ既に広く臨床で用いられており、安全に実施することが可能である。評価は全て対象者1名に対して測定者1名が常に付き添う形で実施し、常に症状や疲労感を確認しながら行う。もし異常所見の出現や、患者自身の中止の申し出があった場合は、速やかに測定は中止する。さらに、異常事態発生に備えて救急用品も常備する。また主治医が院内に勤務し、かつ直ぐに連絡が取れる体制で実施するものとする。  尚、本研究は、事故が発生しないように万全を期して実施し、基本的には医療費の追加は発生しないが、万が一医療費の追加が必要な事態が生じた場合は、誠意を持って対応する。上記については、評価実施に対する患者の精神的負担感を軽減するために、口頭及び書面にて十分に説明する。   - その他（通常診療と研究の位置づけ、参加の自由意思について）   本研究で行われる運動療法の介入と介入前後の身体機能の測定は、通常の透析治療に加えて実施される。カルテ情報に関しては、全て通常診療として実施される医学的な治療と検査の範囲内の項目であり、研究のために追加で測定されない。上記については研究の口頭よび書面（資料①②）で十分に説明の後、同意を頂く。  測定の実施およびカルテ閲覧については、患者様の自由意思によって拒否、もしくは中断することができる旨を、書面と口頭にて説明し、拒否による通常の治療への影響は全く無いことを説明する。 | | | |
| ９　研究成果の公表について（発表の方法・時期・発表機関あるいは場所）  本研究の成果は，関連学会(日本透析医学会、日本腎臓リハビリテーション学会等)での発表又は各種学術誌への投稿にて公開する。発表に際して、個人情報に関する情報は全て匿名化して公表する。 | | | |
| 10　本研究に係る研究費について該当する□にレを入れる。  　□学内共同研究費　　□地域貢献事業研究費　　□その他（　　　　　　　　　　　　　） | | | |
| 11　COIについて該当する□にレを入れる。必要な報告がある場合は、資料を添付する。  　□必要な報告がある　　　　☑必要な報告はない | | | |
| 受付番号 | 受付年月日 | 認証番号 | 判定日 |
|  |  |  |  |

Application for Ethical Review

Seirei Christopher University

Chairperson of the Ethics Committee

Applicant

Institution　Seirei Christopher University

Student ID

Name 　　　　Hiroki Yabe

Academic advisor (if the applicant is a graduate student)

Institution

Name

| １　Study title  Effect of intradialytic exercise on Physical Function in Elderly Hemodialysis Patients: A Randomized Controlled Trial | | | | | |
| --- | --- | --- | --- | --- | --- |
|  | | | | | |
| Name | Institusion | Type of training course attended※ | Details of shared tasks | CITI Japan E-learning certificate number | |
|  |  |  |  | RCR（No）／Fixed day | HSR（No）／Fixed day |
| ２　Corresponding researcher  　　Hiroki Yabe | Seirei Christopher University | ☑CITI elearning  ☑Seminars and workshop | ☑General research work  ☐( ) | ＃6576446  2016. 8. 1. | ＃7228378  2016.8.2 |
|  |  |  |  |  |  |
|  |  |  |  |  |  |
|  |  |  |  |  |  |
|  |  |  |  |  |  |
|  |  |  |  |  |  |
|  |  |  |  |  |  |
|  |  |  |  |  |  |
|  |  |  |  |  |  |
|  |  |  |  |  |  |

| ４Research background  Rehabilitation for elderly hemodialysis patients with reduced physical function and mobility is inadequate. Most dialysis patients have skeletal muscle atrophy associated with protein-energy wasting (PEW) and uremic sarcopenia. Their muscle strength and muscle mass were declined, and physical performance is severely impaired.  Exercise therapy during hemodialysis treatment has a higher continuation rate than other forms of exercise and is considered effective in patients who have difficulty securing time for exercise. Some meta-analysis provides clear evidence that aerobic exercise and resistance training during dialysis can improve muscle strength and exercise tolerance. However, all the randomized controlled trials (RCTs) extracted in this meta-analysis were conducted in dialysis patients who were young or middle age and had preserved physical function. In fact, in a meta-analysis of exercise therapy for elderly hemodialysis patients in 2017, only one RCT on exercise therapy for elderly hemodialysis patients was applicable (Matsuzawa 2017). Currently, it is unclear whether exercise therapy implemented during dialysis treatment for elderly hemodialysis patients improves their physical function.  According to a 2012 report by the Statistical Survey Committee of the Japanese Society for Dialysis Therapy, the largest number of dialysis patients in Japan, both male and female, are between 75 and 80 years of age, and many dialysis patients over 75 years of age require some assistance with ADL. In addition, the number of falls is high among elderly dialysis patients, and their fragile bones can easily fracture, leading to a vicious cycle of further decline in ADL. In order to break this vicious cycle, it is necessary to take measures to improve muscle strength and movement ability, and the establishment of evidence for rehabilitation of elderly dialysis patients is an urgent issue.  Therefore, the purpose of this study was to investigate whether resistance exercise and aerobic exercise during hemodialysis can improve physical function in elderly hemodialysis patients.  Research Objective  To examine whether exercise therapy during a dialysis treatment effectively improves muscle strength, muscle mass, and physical function in elderly dialysis patients by RCT.  Method of the study   - Facility for conducting clinical research: Sanaru Sun Clinic - Period of clinical research: After approval by the Ethics Committee - 1 year - Design: RCT (parallel comparison study)   Subjects  About 100 maintenance hemodialysis patients (50 in the intervention group and 50 in the control group) will recruit. The intervention and control groups will be randomly assigned using a random number table from among patients who meet the criteria. The inclusion and exclusion criteria are shown below.  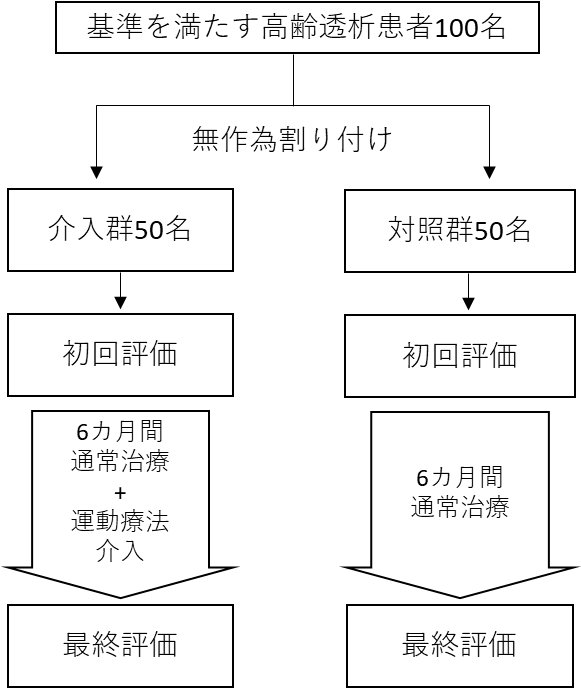  Fig. Study flow  ＜Inclusion criteria   - age ≥ 70 years - a history of hemodialysis for the previous ≥6 months - undergoing hemodialysis for 4 hours, three times per week.   ＜Exclusion criteria＞   - The exclusion criteria were the presence of a physical disability or severe orthopedic problems - A history of stroke within the past 6 months - Recent hospitalization within the past 3 months - Non-ambulatory status - Dementia with an inability to perform exercise and assessment - Acute or chronic medical conditions that would preclude assessment of the outcome measures or performance of an exercise   Methods  Patients randomized to the exercise group were offered 6 months of supervised, individually tailored exercise training three times a week. Each training session was performed during the first 2 hours of each hemodialysis session. The patients underwent hemodialysis and performed each exercise while in the supine position. Each training session began with a 5-minute warm-up period involving stretching exercises. Only lower-extremity exercises were performed to allow temporary vascular catheter access.  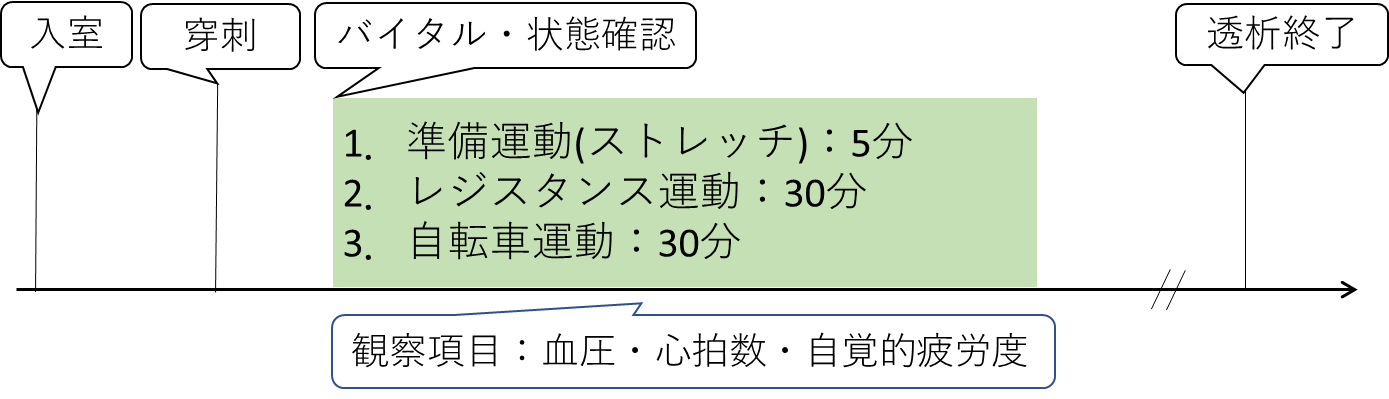  Fig. Exercise protocol   - Preparatory exercises (5 minutes)   Perform self-stretching exercises for hip flexion, internal and external rotation, knee flexion and extension, and plantar dorsiflexion of the ankle joint. Each exercise should be performed 10 times × 1 set.  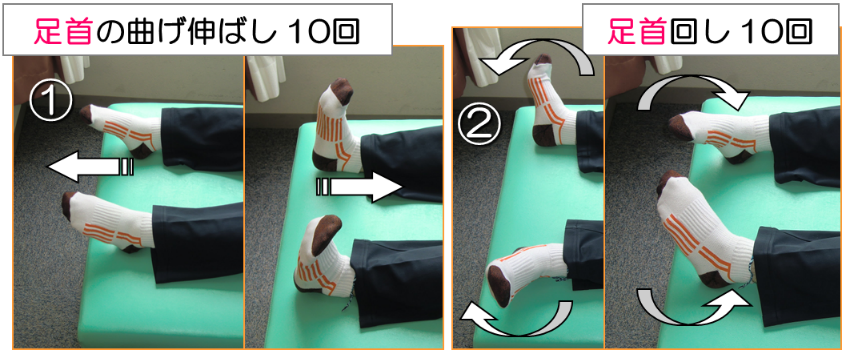  Fig, Examples of preparatory exercise   - Aerobic exercise   The aerobic exercise program specifically targeted aerobic capacity and consisted of ergometer cycling (TerasuErgoⅡ, ShowaDenki, Osaka, Japan) for 20 minutes. The exercise intensity was adjusted to a target Borg score of 13. The aerobic exercise protocols complied as described in previous studies.  Fig. Examples of aerobic exercise   - Resistance exercise   The following four types of resistance exercises were performed using an elastic tube (TheraBand Resistance Band Loops, THERABAND, Akron, OH, USA): leg extension, straight leg raise, hip abduction, and hip flexion. The exercise intensity was adjusted by the tube stiffness to achieve a target Borg score of 13. Three sets were performed, each consisting of 10 repetitions.  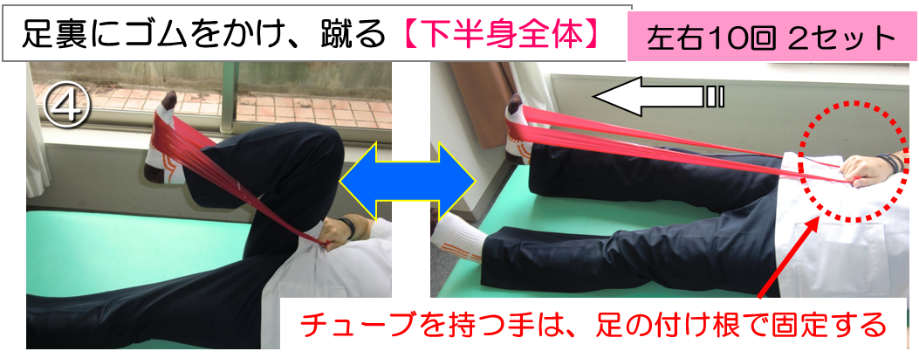  Fig. 　Examples of Resistance exercise  Outocome  As the main outcome, grip strength, knee extensor strength, 10 m walking speed, and Short Physical Performance Battery (SPPB) will be measured as physical functions. Grip strength will be measured in a standing position using a digital grip strength meter (T.K.K5401, Takei Equipment). 2 measurements will be taken on each side, and the maximum value will be recorded. Knee extension muscle strength was measured twice with a manual muscle strength meter (Mobie, Sakai Medical), and the maximum value was recorded.  In addition, height, weight, dry weight, medical history, medication status, laboratory data will be collected from the patient's medical record as basic information.  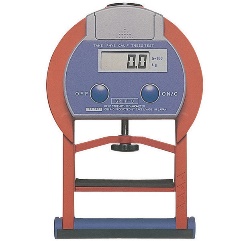  Hand held dynamometer  Mobie TK-11538  Digital Grip Strength Tester T.K.K5401  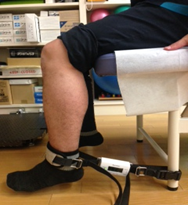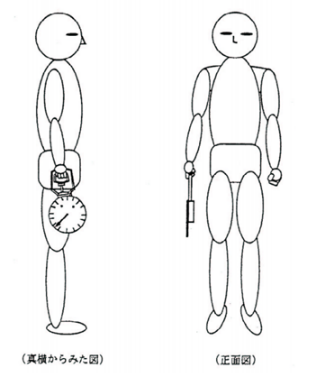  Fig. 　Measurement equipment  ・　Research facilities and measurement locations  　The study will be conducted in the examination room and dialysis room of Sanaru Sun Clinic.  The organizational structure of the research  Hiroki Yabe, the principal investigator of the study, will be responsible for planning the study, checking the study's progress, planning the intervention methods, analyzing the overall data, and writing the paper.  The research collaborators will be responsible for risk management of the study, measurement of the study data, and implementation of the exercise therapy and providing advice on data analysis, and writing the paper.  Table Research collaboration and roles   \| 役割 \| 氏名 \| 所属、役職、職種 \| 研究の分担 \| \| --- \| --- \| --- \| --- \| \| 代表者 \| 矢部広樹 \| 聖隷クリストファー大学、助教、理学療法士 \| 研究計画の立案、研究の進捗確認、データ解析、論文執筆 \| \| 協力者 \| 畦倉久紀 \| さなるサンクリニック、理事長、医師 \| リスクマネージメント \| \| 協力者 \| 山口慶子 \| さなるサンクリニック、総看護師長、看護師 \| データ測定、運動療法の実施 \| \| 協力者 \| 石川有美子 \| 名港共立クリニック　看護主任、看護師 \| データ測定、運動療法の実施 \| \| 協力者 \| 河野健一 \| 国際医療福祉大学、講師、理学療法士 \| データ解析の補助、論文執筆の補助 \| | | | |
| --- | --- | --- | --- | --- | --- | --- | --- | --- | --- | --- | --- | --- | --- | --- | --- | --- | --- | --- | --- | --- | --- | --- | --- | --- | --- | --- | --- |
| ５　Research subjects and selection method  About 100 patients on maintenance hemodialysis at the Sanaru Sun Clinic, the cooperating research facility, will be included in the study, and the inclusion criteria will be as follows.  ＜Inclusion criteria   - age ≥ 70 years - a history of hemodialysis for the previous ≥6 months - undergoing hemodialysis for 4 hours, three times per week.   ＜Exclusion criteria＞   - The exclusion criteria were the presence of a physical disability or severe orthopedic problems - A history of stroke within the past 6 months - Recent hospitalization within the past 3 months - Non-ambulatory status - Dementia with an inability to perform exercise and assessment - Acute or chronic medical conditions that would preclude assessment of the outcome measures or performance of an exercise   ＜Explain the inclusion and exclusion criteria to the attending physician.   1. Explain the inclusion and exclusion criteria to the attending physician and ask him/her to select a candidate. 2. The attending physician will explain the research to the candidate orally and in writing. 3. At that time, the attending physician should fully explain that the cooperation in the research is voluntary, that the consent for participation can be withdrawn at any time, and that there will be no disadvantage or hindrance if the patient does not agree to participate or if the research is suspended. Also, obtain consent from the patient to allow the person in charge to explain the research. 4. For patients who meet the criteria for inclusion and who have received permission from the attending physician and consent from the patient for the explanation, a request for the study will be made orally and in writing and to the patient by the principal investigator or a research collaborator. The explanation will be given in the dialysis room, and if consent is obtained after the explanation, a consent form and a withdrawal of consent form will be handed to the patient. 5. Consent forms will be collected at a later date, after allowing at least one week for consideration, at a collection box set up at the clinic reception desk, with the subject's name and date of consent written on the form. Two copies of the consent form will be prepared, one for the subject and one for the researcher, and each person will keep one copy. The consent forms will be placed in an envelope and collected in the collection box in a manner that protects personal information.   Location: Sanaru Sun Clinic  Explanation of the study: Examination room, Dialysis room  Implementation of exercise therapy: Dialysis room  Measurement of data: Waiting room before dialysis | | | |
| ６　Benefits and Disadvantages of Research  ＜Benefits  The subject will know his or her physical functions by undergoing a physical function assessment. In addition, they will be able to improve their physical functions through exercise therapy.  ＜Disadvantages  The disadvantage of this study is that it requires some fatigue and time constraints due to the implementation of the exercise therapy, time constraints necessary for the physical function evaluation, and some effort and fatigue due to the evaluation. As for time constraints, each exercise session takes about 60 minutes from preparation to completion and continues three times a week for six months. The motor function assessment takes about 30 minutes per session. | | | |
| ７　Reason for application  We would like to review the following items to determine whether ethical considerations are sufficient   - Is the consideration for patients in researching groups appropriate? - Is the method of seeking understanding from the research subjects appropriate? - Is the method of obtaining consent from the research subjects appropriate? - Is the method of obtaining consent from the research subjects appropriate? - Are personal privacy and personal information managed appropriately? - Are personal privacy and personal information appropriately managed? - Is safety appropriately ensured? | | | |
| ８　Ethical considerations in research  Is it appropriate to consider the patients when conducting the study in groups?  The control group will be given verbal and written explanations that they will receive the same exercise therapy intervention as the intervention group if they wish after the 6-month study period and that the effects of exercise will be the same.   - Is the method of seeking understanding from the research subjects clearly defined?   In advance, the research subject should be asked by the attending physician for consent for the researcher to explain the research. I consent for the research's explanation, a request for participation in the research is made using an explanatory document and a request document. In the explanatory document and the purpose of the research, the flow of the research, methods of exercise and evaluation, the safety of exercise, and others should be explained in detail as matters to be actually requested to the research subjects. The explanatory document should use charts, illustrations, and simple terms to make it easy for the subjects to understand the explanation. In the requested document, the purpose of the research, methods, benefits, and disadvantages, measures against anticipated disadvantages, free will to participate in the research and withdrawal of consent, handling of personal information, Publication of research results, and contact points for questions about the research should be explained orally and in writing, respectively.   - Is the method of obtaining consent from the research subjects clearly defined?   Obtain consent from research subjects in the following order   1. Obtain consent for the attending physician to explain the research to the subject. 2. For patients who have given their consent to the explanation of the research, the principal investigator and collaborators will explain the research to the patients orally and in writing. If consent is obtained, hand them a consent form and a withdrawal of consent form. 3. Consent forms will be collected later, after allowing at least one week for consideration, at a collection box set up at the clinic reception desk, with the name and date of consent written on the form after understanding the explanation. The consent form will be placed in an envelope and collected in the collection box in a manner that protects personal information. 4. Two copies of the consent form should be prepared, one for the subject and one for the researcher, and each person should keep one copy.   The withdrawal of consent form will be processed according to the following procedure.   1. At the time of explaining the research, it will be explained that the consent for participation in the research can be withdrawn at any time, and the withdrawal form will be handed together with the consent form. 2. The withdrawal form can be collected at any time from the collection box located at the clinic reception desk. The consent form will be placed in an envelope and collected in the collection box in a way that protects personal information. 3. Research collaborators will check the collection box every morning to collect and receive the consent withdrawal form. 4. Two copies of the withdrawal form will be prepared, one for the subject and one for the researcher, and each will keep one copy. 5. The principal investigator will delete all the subject data who submitted the withdrawal form by shredding paper media and electronic media without leaving any data on the computer.  - Are the privacy and personal information of individuals protected?   Data obtained in the course of research will be treated strictly as personal information, and research data will be processed to prevent the identification of individuals. Measurement data will be entered into a database after anonymization. The data will be anonymized and entered into a database. All response sheets, along with the evaluation result forms, will be kept in a locked vault and stored strictly for 5 years from the study's start date. When the study results are presented at conferences at each facility, they will be presented in a manner that prevents the identification of individuals. The electronic data of the evaluation results will be managed with a password. Electronic data will be managed on a dedicated computer and dedicated external storage media, and the computer will be kept separate from the online environment at all times. When destroying data, paper media should be shredded, and electronic data should be erased using data termination software so that no data remains in the computer. Research collaborators will be able to view the data only from the computers dedicated to this research and not copy and use the data.   - Are safety considerations taken into account?   It has been shown that exercise therapy can be performed safely during the first two hours of hemodialysis when the circulatory system is stable. Exercise therapy for hemodialysis patients is recommended in the guidelines of the American College of Sports Medicine. The exercise program in this study is also recommended by the American College of Sports Medicine and other organizations as a method of exercise therapy for hemodialysis patients and the elderly and can be performed safely. If there are any abnormal findings or if the patient himself requests to stop the exercise, the exercise will be stopped immediately. In addition, emergency supplies should be kept on hand in case of abnormalities. In addition, the study should be conducted in a system where the attending physician works in the hospital and can be immediately contacted.  The physical function assessment to be used in the study has been established in geriatrics and is already widely used in clinical practice, so it can be conducted safely. All assessments will be conducted with one measurer accompanying each subject at all times while constantly checking for symptoms and fatigue. If any abnormal findings appear or the patient himself requests to stop the measurement, the measurement should be stopped immediately. In addition, emergency supplies are always available in case of abnormalities. In addition, the study should be conducted in a system where the attending physician works in the hospital and can be contacted immediately.  In addition, this study will be conducted with all possible precautions to prevent accidents from occurring, and in principle, no additional medical expenses will be incurred. The above information will be fully explained orally and in writing to reduce the patient's mental burden of the evaluation.   - Others (positioning of the study about regular medical treatment and free will to participate)   The exercise therapy intervention and the measurement of physical functions before and after the intervention in this study will be conducted in addition to the regular dialysis treatment. As for the medical record information, all items will be within the scope of medical treatment and examination performed as part of regular medical care, and no additional measurements will be taken for the study. The above will be fully explained orally and in writing, and consent will be obtained.  It will be explained both orally and in writing that the patient may refuse or suspend the measurement and access to medical records at his/her own free will and that the refusal will not affect the normal treatment at all. | | | |
| ９　Publication of research results (method, timing, and institution or place of presentation)  The results of this study will be made public through presentations at relevant academic societies (e.g., the Japanese Society of Dialysis Therapy, the Japanese Society of Renal Rehabilitation, etc.) or submissions to various academic journals. All personal information will be anonymized before Publication. | | | |
| 10　Check the appropriate box regarding the research expenses for this research.  　□学内共同研究費　　□地域貢献事業研究費　　□その他（　　　　　　　　　　　　　） | | | |
| 11　 Check the appropriate box regarding COI. If there are required reports, attach documents.  　Required report ☑Not required report | | | |
| Reception number | Reception date | Certification number | Judgment date |
|  |  |  |  |
